# Zilebesiran: an RNA interference agent—its need and potential to transform hypertension treatment

**DOI:** 10.1186/s43044-026-00724-9

**Published:** 2026-03-01

**Authors:** Sajeet Verma, Akshyaya Pradhan, Prashant Thandi

**Affiliations:** Department of Cardiology, King George’s Medical University, Lucknow, India

## Abstract

**Background:**

Hypertension remains the leading modifiable risk factor for cardiovascular morbidity and mortality worldwide, yet nearly half of patients fail to achieve target blood pressure (BP) despite the availability of multiple antihypertensive agents. Non-adherence, therapeutic inertia, and complex dosing regimens continue to undermine treatment effectiveness. The renin–angiotensin–aldosterone system (RAAS) is central to BP regulation, and long-term blockade of its components reduces cardiovascular risk. However, current therapies require daily adherence. Zilebesiran, a novel RNA interference (RNAi) therapeutic, represents an innovative approach by silencing hepatic angiotensinogen (AGT) synthesis, offering sustained RAAS suppression with infrequent dosing.

**Main text:**

Zilebesiran is a GalNAc-conjugated small interfering RNA (siRNA) that targets AGT mRNA in hepatocytes via asialoglycoprotein receptor–mediated delivery. This mechanism leads to durable reductions in circulating AGT and downstream angiotensin II, providing consistent BP lowering for up to 6 months after a single subcutaneous injection. Phase I and II trials demonstrated > 90% AGT suppression and clinically significant reductions in 24-h systolic BP (− 10 to − 27 mmHg), with favorable safety and tolerability. The KARDIA-1 and KARDIA-2 studies confirmed zilebesiran’s sustained efficacy as monotherapy and as an adjunct to standard antihypertensive agents, without major renal or electrolyte disturbances.

**Conclusions:**

Zilebesiran may redefine therapy in hypertension management through twice-yearly dosing that enhances adherence, ensures sustained BP control, and may reduce cardiovascular risk. Ongoing Phase III trials (ZENITH and KARDIA-3) will clarify its long-term efficacy, safety, and applicability across diverse populations, potentially establishing RNAi therapeutics as a new frontier in chronic hypertension treatment.

## Background

Uncontrolled hypertension (HTN) is a critical contributor to major cardiovascular events and impaired renal function, standing as the leading preventable factor for cardiovascular deaths worldwide. Despite the availability of effective treatments, nearly half of patients with HTN fail to meet the blood pressure (BP) targets recommended by guidelines [1, 2]. Even patients with apparently stable BP during routine check-ups may have substantial BP variability between follow-ups, which is associated with an ongoing risk for cerebro–cardiovascular events [3, 4]. Two intertwined problems undermine HTN control: difficulty sustaining daily adherence to multiple medications and clinical inertia in escalating therapy [5].

In addition to genetic predisposition and pharmacologic non-adherence, sedentary lifestyle and unhealthy diet are becoming key contributors to uncontrolled HTN. Physical inactivity promotes weight gain, sympathetic overactivity, and endothelial dysfunction, thereby increasing BP. Regular aerobic exercise reduces systolic BP by 5–8 mmHg, which is why it is considered a therapeutic factor [6]. Similarly, suboptimal nutrition, characterized by high sodium intake, low potassium consumption, and excessive intake of processed and high-fat foods, significantly increases HTN risk. Evidence from large cohort studies, such as the PURE [7] and INTERSALT [8] studies, demonstrates strong associations between unhealthy dietary patterns, physical inactivity, and elevated BP levels. Addressing these modifiable lifestyle factors is therefore essential for long-term BP control and cardiovascular risk reduction.

As the dominant modifiable driver of vascular and organ damage, elevated BP significantly increases risks for cerebral infarction, cardiac dysfunction, renal deterioration, and plaque-driven arterial disease. Notably, a mere 5 mmHg drop in systolic blood pressure (SBP)—achievable through lifestyle or pharmacological intervention—translates to a near 10% decline in incident cardiovascular events among asymptomatic individuals, escalating to an 11% protective effect in patients with established disease [9]. Similarly, evidence from large meta-analyses [10] shows that a 10 mmHg reduction in office SBP is associated with approximately a 20% reduction in major cardiovascular events. However, these data are primarily based on office BP measurements, which differ from ambulatory (ABPM) and home BP monitoring (HBPM) [11]. While all methods aim to assess average BP and correlate with cardiovascular outcomes, ABPM and HBPM provide greater accuracy and prognostic value. The white-coat or masked HTN effect can influence office BP, whereas ABPM captures 24-hour BP variability, including nocturnal patterns, offering superior prediction of target-organ damage and cardiovascular risk. HBPM provides reproducible readings in a home environment and aligns more closely with ABPM data. Therefore, while office BP–based reductions remain the benchmark for outcome trials, out-of-office measurements may offer a more reliable reflection of true BP control and cardiovascular risk. This dose–response relationship emphasizes that graduated BP control, even at marginal levels, carries measurable clinical significance in mitigating the global burden of hypertensive complications. Although antihypertensive treatment is proven to lower cardiovascular risk and improve longevity, BP control, treatment persistence, and adherence remain suboptimal. A meta-analysis of 27 million hypertensive patients revealed that 27–40% fail to adhere to their prescribed therapy [12]. Therapeutic inertia is another major factor contributing to poor BP control among hypertensive patients. According to the 2023 European Society of Hypertension (ESH) guidelines, most patients should begin antihypertensive therapy with a combination of two medications, ideally as a single-pill combination (SPC) [13, 14]. Poor adherence to antihypertensive treatment stems from factors such as lack of awareness, complicated pill schedules, intricate dosing protocols, economic constraints, low motivation, limited healthcare access, and concerns about side effects [15].

## Aim of this study

To review and synthesize current clinical evidence on zilebesiran, focusing on its mechanism of action, antihypertensive efficacy, safety, and durability of BP control, and to evaluate its potential to address key limitations of conventional antihypertensive therapy—particularly poor adherence and the need for daily dosing—while highlighting its prospective role in transforming long-term HTN management.

### Methods

A systematic literature search was performed across PubMed, Scopus, Web of Science, and ClinicalTrials.gov to identify studies evaluating zilebesiran and other small-interfering RNA (siRNA)–based approaches for the treatment of HTN. The search encompassed articles published between January 2012 and June 2025. Search terms included “zilebesiran,” “hypertension,” “siRNA therapy,” “angiotensinogen inhibition,” and “RNA interference,” used alone or in combination.

Eligible studies comprised preclinical studies and clinical trials assessing efficacy or safety. Conference abstracts were included only if full-text data were unavailable and methodological details were adequate. Reviews, editorials, duplicates, non-HTN studies, and non-English articles were excluded. Study selection involved title/abstract screening followed by full-text review, prioritizing human clinical studies.

No formal risk-of-bias or quality assessment was performed due to heterogeneous designs and early-phase evidence; study quality was evaluated descriptively, and findings should be interpreted with caution.

## RAAS in HTN and cardiovascular protection

The renin–angiotensin–aldosterone system (RAAS) is a key regulator of BP, and its activation initiates physiological processes that increase vascular resistance, expand plasma volume, stimulate aldosterone secretion, and disrupt sodium–potassium balance. Central to this system is angiotensin II (Ang II), a potent vasoconstrictor formed from angiotensin I (Ang I) by angiotensin-converting enzyme. Ang I is generated when renal renin cleaves liver-derived angiotensinogen (AGT). Together, these tightly regulated steps highlight the essential role of RAAS in cardiovascular homeostasis and its contribution to HTN when dysregulated [16].

RAAS inhibitors effectively lower blood pressure and reduce cardiovascular risk. A large meta-analysis of 158,998 patients showed that ACE inhibitors reduced all-cause mortality by 10%, with benefits emerging after 3–5 years of sustained therapy.

The Blood Pressure Lowering Treatment Trialists’ Collaboration (BPLTTC) [17] demonstrated that ACEI-based regimens reduced all-cause mortality by approximately 10% and major cardiovascular events by about 20% across diverse patient populations. Similarly, the HOPE trial reported significant mortality and morbidity reductions with ramipril over a mean follow-up of 5 years. The EUROPA trial [18] further confirmed these findings, showing a 20% reduction in cardiovascular death and morbidity with perindopril over 4.2 years of follow-up.

Collectively, these studies establish that the mortality benefit of ACEIs becomes evident with sustained treatment over several years, reflecting both direct BP-lowering and vascular protective effects. These results underscore the critical role of RAAS inhibition in managing HTN and reducing the burden of cardiovascular morbidity and mortality [19].

RAAS blockade enhances endothelial function by reducing oxidative stress, increasing nitric oxide bioavailability, and suppressing inflammatory signaling. ACE inhibitors and ARBs limit vascular inflammation, thrombosis, and remodeling, while myocardial Ang II and aldosterone inhibition reduces hypertrophy, fibrosis, and adverse cardiac remodeling.

Collectively, these mechanisms explain why RAAS inhibition lowers cardiovascular morbidity and mortality rates in patients with HTN, heart failure, or ischemic heart disease, even when BP reduction is modest. Thus, the cardioprotective benefits of RAAS inhibitors extend well beyond simple BP control, reflecting their broad anti-inflammatory, anti-fibrotic, and vasculoprotective actions [20].

### siRNA-based drug in HTN (mechanism of action)

ACEIs are widely regarded as the gold-standard treatment for managing HTN. To enhance patient compliance, an innovative strategy focuses on minimizing the number of doses required per day through advanced RNA-based technology. This novel approach works by silencing the AGT gene in the liver, leading to a significant reduction in the production of Ang I and Ang II. By lowering Ang II levels, this strategy effectively disrupts signaling through angiotensin receptor type 1 (AT1R) and type 2 (AT2R). As a result, it offers the potential for sustained BP control over an extended period, addressing challenges associated with daily medication adherence and improving long-term treatment outcomes.

As an innovative treatment modality, zilebesiran harnesses RNA silencing mechanisms specifically engineered to inhibit hepatic AGT production. By targeting AGT, the primary precursor of angiotensin peptides that regulate BP, zilebesiran emerges as a novel strategy for HTN treatment.

**Fig. 1:**
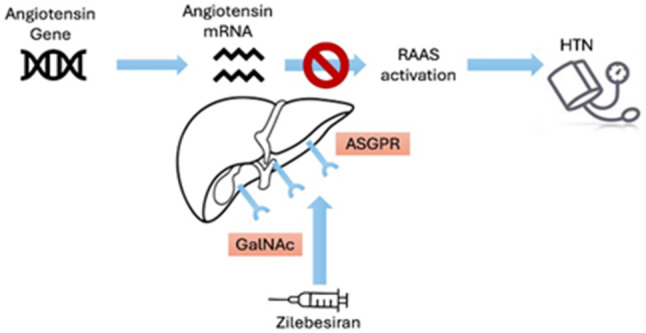
Mechanism of action of zilebesiran in reducing hypertension

The use of advanced technology has enabled the development of small interfering RNA (siRNA) molecules with low immunogenicity, linked to N-acetylgalactosamine (GalNAc) for targeted delivery. Zilebesiran, a GalNAc-conjugated siRNA, selectively binds to the asialoglycoprotein receptor (ASGPR), a protein predominantly expressed on the surface of hepatocytes. This receptor facilitates rapid transport of the siRNA into endosomes, which serve as reservoirs for messenger RNA (mRNA) knockdown and allow siRNA recycling. Knockdown of AGT mRNA leads to a significant reduction in AGT production, thereby suppressing renin-mediated angiotensin synthesis (Fig. 1).

Although hepatic AGT is the predominant circulating source, extrahepatic sites—especially the kidney—retain AGT expression despite hepatic suppression, highlighting their potential independent contribution to local renin–angiotensin activity. This targeted approach can result in BP lowering that lasts for several months, enabling dosing intervals of 3–6 months [21].

Since zilebesiran inhibits hepatic AGT production, the amount of Ang I and II in the blood decreases significantly, indicating effective upstream inhibition of the renin–angiotensin system. Conversely, plasma renin concentration and activity normally increase as a compensatory response to reduced Ang II-mediated feedback suppression. These dynamic changes are also monitored and may provide valuable mechanistic insight into the extent and duration of RAS suppression, and could contribute to explaining the sustained antihypertensive effect despite short-term changes in renin activity.

### Safety and efficacy evidence from clinical trials

In a phase I randomized study by Huang et al., 60 participants with mild-to-moderate HTN received single subcutaneous doses of zilebesiran (100 mg or 200 mg). The therapy demonstrated potent and durable effects, suppressing serum AGT by more than 90% for up to 12 weeks. By week 8, it also elicited a clinically significant 10 mmHg reduction in 24-hour SBP. Importantly, zilebesiran exhibited a favorable safety profile, with no life-threatening reactions, electrolyte imbalance, or clinically relevant alterations in renal function [22].

Another phase I trial conducted by Taubel et al. investigated the effects of zilebesiran in comparison with irbesartan in 20 patients with HTN and obesity. Participants received either zilebesiran (800 mg administered on days 1 and 85) or irbesartan (150 mg daily). The study demonstrated that zilebesiran achieved a profound reduction in serum AGT levels, decreasing them by 99% from weeks 4 to 24, whereas irbesartan showed no significant impact on AGT levels. In terms of BP control, zilebesiran led to a substantial reduction in SBP of − 27 ± 8 mmHg, outperforming irbesartan, which resulted in a reduction of − 19 ± 6 mmHg. Reassuringly, zilebesiran showed no concerning side effects, with no clinically significant adverse events observed in the trial [23]. It should be noted, however, that the study did not include specific assessments of adherence to irbesartan therapy, as the drug was administered in an open-label, non-randomized design, which may limit direct comparisons regarding compliance-related efficacy differences between the two treatments.

The unique mechanism of action of zilebesiran likely accounts for the low incidence of severe orthostatic hypotension, even with profound suppression of circulating AGT. As a liver-directed small interfering RNA (siRNA), zilebesiran selectively silences hepatic AGT mRNA while leaving extrahepatic sources—such as those in the kidney, brain, and heart—largely intact. Such selective targeting preserves local RAAS activity, which helps maintain vascular tone and autonomic regulation and thus avoids sudden drops in postural BP. Moreover, zilebesiran provides upstream and sustained blockade of the RAAS, resulting in a gradual, steady, and prolonged reduction in BP that persists for several months after a single dose. This contrasts with the rapid and unstable BP changes that are occasionally observed with daily oral antihypertensive treatments and that predispose patients to orthostatic events.

In a separate phase I trial led by Desai et al., (Table 1) zilebesiran was evaluated in a larger cohort of 107 patients with HTN. This study was conducted following a washout period to ensure that baseline measurements were not influenced by prior antihypertensive treatments. The trial aimed to further assess the efficacy, safety, and tolerability of zilebesiran in a broader patient population, providing additional insight into its potential as a novel therapeutic option for HTN management. This trial demonstrated the promising role of zilebesiran in significantly reducing AGT levels and BP while maintaining a favorable safety profile.

Single doses (10–800 mg) were studied, with the 800 mg dose showing a > 90% reduction in serum AGT and a dose-dependent 24-hour BP reduction. The 800 mg group exhibited marked antihypertensive effects at 24 weeks, with SBP and diastolic BP decreasing by 22.5 and 10.8 mmHg, respectively. Zilebesiran showed a good safety profile, with no hypotension, hyperkalemia, or kidney-related issues, suggesting that twice-yearly dosing could effectively control BP in some patients [24].

**Fig. 2:**
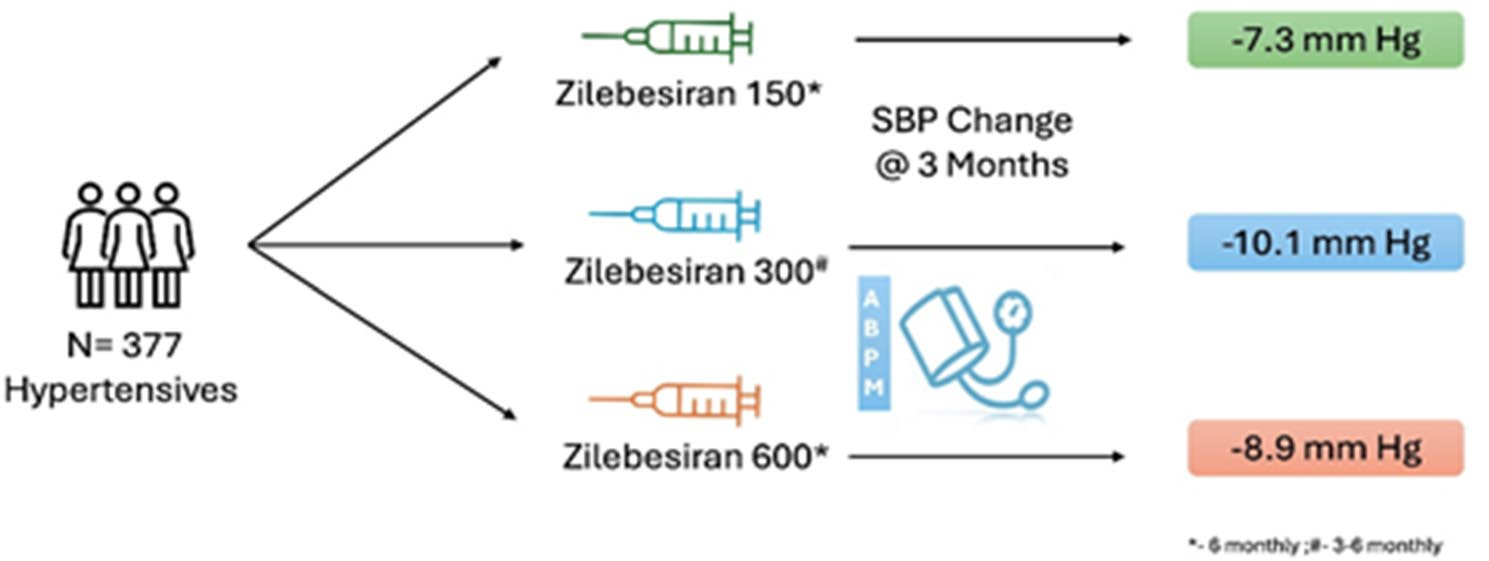
- Effect of zilebesiran on systolic blood pressure in hypertensive patients at 3 months in the KARDIA-1 study

The KARDIA-1 trial **(**Fig. 2), a randomized, double-blind, placebo-controlled phase 2 study conducted between July 2021 and June 2023, investigated the efficacy and safety of zilebesiran in adults with mild-to-moderate HTN, defined as a daytime SBP ranging from 135 to 160 mmHg. The study was initiated after a 2- to 4-week washout period to eliminate the influence of prior antihypertensive medications, ensuring an unbiased assessment of zilebesiran’s effects [25].

Participants received subcutaneous zilebesiran (150, 300, or 600 mg every 6 months, or 300 mg every 3 months) or placebo for 6 months (Table 1). No other antihypertensives were allowed until month 3, with oral medications (if any) stopped at month 5 to assess zilebesiran’s isolated effect at month 6. In total, 377 patients (302 receiving zilebesiran and 75 receiving placebo; 24.7% Black, 44.3% women; mean age 57 years) were analyzed. At month 3, changes in 24-hour mean ambulatory systolic BP were − 7.3 mmHg (zilebesiran 150 mg every 6 months), − 10.0 mmHg (300 mg every 3 or 6 months), − 8.9 mmHg (600 mg every 6 months), and + 6.8 mmHg (placebo). Reductions were consistent over 24 h and persisted through month 6. The study demonstrated that zilebesiran produced a significant antihypertensive effect (approximately 10 mmHg reduction in 24-hour SBP) with an acceptable safety profile. Common adverse effects included mild, transient injection-site reactions (6.3%) and hyperkalemia (5.3%). No evidence of impaired kidney or liver function was detected (Fig. 2).

The KARDIA-2 (Table 1) study was a rigorously designed, double-blind, placebo-controlled clinical trial that evaluated the efficacy of zilebesiran in managing BP among patients with HTN that remained uncontrolled despite standard antihypertensive therapy. The trial enrolled 672 participants, who were initially treated with one of three standard medications: indapamide 2.5 mg, amlodipine 5 mg, or olmesartan 40 mg daily. Following a run-in period to ensure baseline stability, participants were further randomized to receive either a 600 mg subcutaneous dose of zilebesiran or placebo in addition to their assigned antihypertensive medication.

The results demonstrated that zilebesiran significantly lowered 24-hour systolic BP over 3 months compared with placebo. Reductions were − 12.1 mmHg in the indapamide group (*p* < 0.001), − 9.7 mmHg in the amlodipine group (*p* < 0.001), and − 4.0 mmHg in the olmesartan group (*p* = 0.036). Notably, the indapamide and amlodipine groups maintained these BP reductions for up to 6 months, highlighting the durability of zilebesiran’s effect when combined with these agents. Office BP measurements at 3 months also showed significant declines of − 18.5 mmHg, − 10.2 mmHg, and − 7.0 mmHg in the indapamide, amlodipine, and olmesartan groups, respectively (*p* < 0.001). Participants tolerated the intervention well, with no deaths or adverse events requiring treatment discontinuation reported.

In summary, zilebesiran demonstrated a meaningful improvement in 3-month BP control among patients with uncontrolled HTN, particularly when used in combination with indapamide or amlodipine, with sustained benefits observed up to 6 months. However, its effect was less pronounced in the olmesartan group. These findings underscore zilebesiran’s potential as a valuable adjunct therapy for improving BP management in patients who do not achieve adequate control with standard antihypertensive regimens [26].

**Table 1:** Comparison of clinical trials evaluating zilebesiran for hypertension management

Study ID	Study type and number of participants (*N*)	Patient group	Control group	Treatment given	Primary endpoint(s) & follow-up duration	Key findings	Limitations
Bakris [25]	Randomized, double-blind clinical trial (394)	Mild–moderate primary hypertension	Placebo	Subcutaneous zilebesiran, single doses 10–800 mg (Part A), 800 mg with low-/high-salt diet (Part B), 800 mg + irbesartan (Part E)	Safety profile, pharmacokinetic and pharmacodynamic characteristics, and effects on 24-hour ambulatory blood pressure at 3 months 3–6 months	A single subcutaneous dose of zilebesiran led to significant and clinically relevant reductions in systolic blood pressure (SBP), which persisted for up to 6 months.	Mainly enrolled mild–moderate hypertension patients. Excluded major comorbidities, and had limited follow-up.
Saxena [26]	Randomized, double-blind clinical trial (667)	Hypertension uncontrolled on therapy	Placebo combined with a conventional antihypertensive drug (Indapamide, Amlodipine, Olmesartan)	Subcutaneous zilebesiran 600 mg combined with a conventional antihypertensive drug (Indapamide, Amlodipine, Olmesartan)	Change in 24-h ambulatory SBP and Effectiveness and tolerability as add-on therapy. 3–6 months	When added to standard therapy, zilebesiran significantly lowered 24-h average ambulatory SBP (primary outcome) and office SBP (key secondary outcome) compared to placebo after 4 months.	Evaluated zilebesiran only as add-on therapy, making it difficult to isolate monotherapy efficacy. Variable response depending on background antihypertensive class.
Desai [24]	Randomized, double-blind clinical trial (84)	Individuals with primary hypertension	Placebo	Single ascending subcutaneous doses (10–800 mg); follow-up up to 24 weeks	Safety profile, pharmacokinetic and pharmacodynamic characteristics, and change in 24-h SBP. 6 months	A single dose of zilebesiran resulted in dose-dependent reductions in both serum angiotensinogen levels and 24-h ambulatory blood pressure, with effects lasting up to 24 weeks.	Single-dose design. Short-to-intermediate follow-up (≤ 24 weeks). Limited insight into long-term safety, chronic dosing effects, and cardiovascular outcomes.

## Limitations

This study has several important limitations. The available evidence is derived from small sample sizes and short follow-up durations, limiting robust conclusions on long-term efficacy and safety. Only one Phase 2 trial evaluated zilebesiran as monotherapy, while others assessed it as an add-on to background antihypertensive therapy, complicating interpretation of its independent BP-lowering effect. Early-phase studies further provided limited mechanistic and clinical insight due to low participant numbers.

Moreover, the study populations largely consisted of patients with mild-to-moderate HTN and few comorbidities, restricting generalizability to higher-risk or more diverse hypertensive groups. Zilebesiran is contraindicated during pregnancy and lactation. Although animal studies showed no fetal harm or placental transfer, these data are insufficient to confirm safety in humans. Renal safety data remain incomplete, with no reporting on albuminuria and insufficient long-term evaluation of eGFR. Finally, while sustained BP reduction has been observed for up to 6 months after a single dose, the long-term consequences of chronic AGT silencing and outcomes following treatment withdrawal remain unclear.

## Clinical implications and future perspectives

Zilebesiran represents an emerging therapeutic strategy in hypertension that targets a well-recognized barrier to effective blood pressure control—suboptimal adherence to long-term pharmacotherapy. By offering infrequent subcutaneous administration, it may reduce daily pill burden and simplify treatment regimens, particularly for patients with complex medication schedules or documented adherence difficulties. However, lifestyle modification and conventional antihypertensive therapies remain the foundation of hypertension management, and the role of zilebesiran should be viewed as complementary rather than substitutive at this stage.

Beyond blood pressure reduction, zilebesiran could potentially offer benefits in selected high-risk populations, although evidence for target-organ protection or cardiovascular risk reduction is not yet available. Ongoing phase 3 trials, including KARDIA-3 and ZENITH, are expected to provide critical data regarding its efficacy, safety, and clinical applicability in resistant hypertension and patients with elevated cardiovascular risk. Until long-term outcome data are available, the clinical use of zilebesiran should be considered investigational, with its ultimate position in hypertension treatment algorithms remaining to be defined.

## Conclusions

Despite the availability of multiple approved antihypertensive therapies, blood pressure control remains suboptimal in a substantial proportion of patients, with poor medication adherence representing a key contributing factor. Zilebesiran, an investigational small-interfering RNA therapy administered at extended intervals, offers a novel pharmacologic approach that may help address adherence-related challenges in hypertension management.

Early-phase clinical trials suggest that zilebesiran is generally well tolerated, with no serious adverse events related to renal function or electrolyte disturbances reported in phase 1 and 2 studies. Laboratory parameters, including serum creatinine, liver enzymes, and potassium levels, demonstrated no clinically meaningful changes over the durations studied. While the infrequent dosing schedule (every 3–6 months) has the potential to reduce cumulative drug exposure and improve adherence, longer-term data are required to confirm the durability of blood pressure lowering, long-term safety, and broader clinical impact. Accordingly, current findings should be interpreted as preliminary and hypothesis-generating rather than definitive.

## Data Availability

No datasets were generated or analysed during the current study.

## Abbreviations

ABPM: Ambulatory blood pressure monitoring
ACEI: Angiotensin-converting enzyme inhibitor
AGT: Angiotensinogen
Ang I: Angiotensin I
Ang II: Angiotensin II
ASGPR: Asialoglycoprotein receptor
AT1R: Angiotensin II type 1 receptor
AT2R: Angiotensin II type 2 receptor
BP: Blood pressure
BPLTTC: Blood pressure lowering treatment trialists’ collaboration
CVD: Cardiovascular disease
ESH: European society of hypertension
HBPM: Home blood pressure monitoring
HTN: Hypertension
INTERSALT: International study of electrolyte excretion and blood pressure
PURE: Prospective urban rural epidemiology study
RAAS: Renin–angiotensin–aldosterone system
RNA: Ribonucleic acid
siRNA: Small interfering RNA
SBP: Systolic blood pressure
SPC: Single-pill combination
ZENITH: Zilebesiran cardiovascular outcome study in hypertension
